# Neuroprotection by Resveratrol in Chronic Cerebral Hypoperfusion: A Study on Synaptogenesis Enhancement and Apoptosis Inhibition

**DOI:** 10.5812/ijpr-162425

**Published:** 2025-09-02

**Authors:** Abdollah Kasaei, Mohsen Forouzanfar, Mojtaba Jafarinia

**Affiliations:** 1 Department of Biology, Marv.C., Islamic Azad University, Marvdasht, Iran

**Keywords:** Chronic Cerebral Hypoperfusion, Resveratrol, Apoptosis, Synaptogenesis, Rat

## Abstract

**Background:**

Chronic cerebral hypoperfusion (CCH) is a key contributor to vascular dementia (VaD) and Alzheimer’s disease. Resveratrol (RSV), a polyphenol with potential neuroprotective properties, may mitigate CCH-induced neuronal damage, but its mechanisms remain unclear.

**Objectives:**

This study investigated RSV’s effects on memory enhancement through synaptogenesis and apoptosis inhibition in the hippocampus in a rat CCH model.

**Methods:**

Forty male rats were randomly divided into four groups: Sham, 2-VO (bilateral carotid artery occlusion), 2-VO+RSV (2.5 mg/kg), and 2-VO+RSV (5 mg/kg). Initial group sizes (n = 10 each) were maintained by replacing deceased animals (2-VO: 7, 2-VO+RSV2.5: 4, 2-VO+RSV5: 6 deaths). The RSV was administered via intraperitoneal injection (ip) for 35 days post-surgery. Cognitive function was assessed using Morris water maze (MWM) and shuttle box tests. Hippocampal mRNA/protein levels of B-cell lymphoma 2-associated X (Bax), B-cell lymphoma 2 (Bcl-2), Caspase-3, Ras homolog family member A (RhoA), Rho-associated coiled-coil containing protein kinase 2 (ROCK2), calcium/calmodulin-dependent protein kinase II alpha (CaMKII-α), and N-methyl-D-aspartate receptor subunit 2B (NMDAR2B) were measured.

**Results:**

The RSV (5 mg/kg) significantly improved spatial memory in the MWM. Also, RSV at doses of 2.5 and 5 mg/kg significantly increased the entrance latency to the dark compartment (P < 0.05 and P < 0.01 vs 2-VO, respectively). There was a downregulation of pro-apoptotic markers (Bax, Caspase-3) and Rho/ROCK gene expressions, and an upregulation of anti-apoptotic Bcl-2 gene expression and synaptic proteins (CaMKII-α, NMDAR2B) after RSV treatment. The RSV at 5 mg/kg significantly reduced the Bax/Bcl-2 ratio compared to the 2-VO group.

**Conclusions:**

The RSV protects against CCH-induced neuronal damage by inhibiting apoptosis and enhancing synaptic plasticity. These findings highlight RSV’s therapeutic potential for vascular cognitive impairment.

## 1. Background

Chronic cerebral hypoperfusion (CCH) is a common mechanism for causing cerebrovascular disorders with pathological processes of vascular dementia (VaD), leading to various neurological impairments (1). Cognitive impairments, such as learning and memory deficits, are closely linked to CCH in vascular cognitive impairment (2). Many studies in this field have reported that apoptosis may play a role in the onset and progression of cognitive impairments resulting from VaD (3). Key elements of apoptosis regulated and expressed in the hippocampus consist of both pro-apoptotic and anti-apoptotic factors (4).

Pro-apoptotic factors such as B-cell lymphoma 2-associated X (Bax) trigger mitochondrial membrane permeabilization, leading to cytochrome c release into the cytosol, ultimately activating Caspase 9 and then Caspase 3, leading to cell death. Some studies have shown that increased activation of Caspase 3 leads to cerebral vascular barrier disruption, glial cell activation, and oxidative stress, ultimately resulting in cognitive impairments (5, 6). The B-cell lymphoma 2 (Bcl-2) protein prevents the release of cytochrome C into the cytosol, thereby blocking apoptosis by inhibiting Bax protein (7, 8).

Other pathological changes that occur during CCH include neuronal and glial loss (9). It is believed that the degradation in the hippocampal Cornu Ammonis 1 (CA1) region contributes to cognitive impairments, especially memory and learning dysfunction (10). Since the majority of patients with VaD suffer from neuropathological impairments, finding effective drugs to identify the mechanisms involved in vascular cognitive decline is essential (11). Neurogenesis, angiogenesis, and synaptogenesis may improve lesions and inhibit apoptosis in VaD (12). Structural and functional aspects of synaptic plasticity serve as the neural substrate for learning and memory formation, which are crucial (13). Synaptic plasticity has been reported to be associated with the number of synapses and changes in synaptic space structurally, while functional synaptic plasticity refers to the increase and decrease in synaptic transmission efficiency (13, 14). It has been identified that cognitive decline, often including forgetfulness and reduced learning and memory abilities, is associated with synaptic dysfunction and impairment (15). Currently, the focus for treating cognitive impairments has been more on preventing excessive brain damage. Recently, multiple studies have reported that plants containing phenols may be a more suitable choice for treating and preventing brain damage in diseases that cause cognitive decline (16).

Resveratrol (RSV) is a natural non-flavonoid polyphenolic compound found in grapes, berries, peanuts, and in the roots and fruits of several plants (17, 18). This compound has multiple effects, including anti-cancer (19), anti-inflammatory (20), and neuroprotective properties (21). It has also been shown that RSV significantly reduces pyramidal cell death in the hippocampus after CCH and prevents memory and learning impairments (22). Numerous studies have reported independent effects of RSV in the treatment of neurological disorders, and it has been hypothesized that RSV, through its neuroprotective effects, can minimize cognitive impairments in neurodegenerative patients. However, to date, no detailed study has been conducted on how RSV affects neurogenesis and synaptogenesis simultaneously to improve lesions resulting from VaD.

## 2. Objectives

While previous studies have highlighted RSV’s neuroprotective potential in cerebral hypoperfusion, its simultaneous effects on synaptic plasticity and apoptosis pathways remain unclear. This study aimed to investigate whether RSV (at doses of 2.5 and 5 mg/kg) could mitigate cognitive deficits in a rat model of CCH by modulating apoptotic markers (Bax/Bcl-2 ratio, Caspase-3), enhancing synaptic proteins [calcium/calmodulin-dependent protein kinase II alpha (CaMKII-α), N-methyl-D-aspartate receptor subunit 2B (NMDAR2B)], and suppressing the Rho/ROCK pathway. We further correlated these molecular changes with behavioral outcomes [Morris water maze (MWM), shuttle box] to establish RSV’s dual role in synaptogenesis promotion and apoptosis inhibition.

## 3. Methods

### 3.1. Experimental Procedures

Adult male Wistar rats with normal weight (200 to 250 g) were sourced from the Islamic Azad University Marodasht Branch's Animal House. During the experiment, the animals were housed in clean cages under controlled conditions: A 12-hour light/dark cycle to regulate their circadian rhythms at a controlled temperature of 22 - 25°C with unrestricted access to food and water. The experiment received ethical approval from the Ethical Committee of Islamic Azad University, Marvdasht Branch, Iran (ethical code: IR.IAU.M.REC.1402.103).

### 3.2. Experimental Designs

The rats (n = 10 per group) were randomly assigned to four distinct experimental groups. The groups included: Sham (subjected to surgical stress without occlusion of the common carotid arteries), CCH (2-VO; common carotid artery occlusion). Twenty rats were subjected to 2-VO and then received RSV for five weeks at doses of 2.5 and 5 mg/kg [2-VO+RSV2.5 and 2-VO+RSV5, intraperitoneal (ip) injection]. To ensure consistent group sizes, deceased animals (2-VO: 7; 2-VO+RSV2.5: 4; 2-VO+RSV5: 6) were replaced with new subjects undergoing identical procedures. Replacement animals were acclimatized for 7 days before data collection. Final analyses included all animals (original+replacements). Rats were stratified by initial weight before randomization to ensure equal distribution (200 - 250 g), and to prevent selection bias, group allocation was concealed from the experimenters until the end of the surgical procedure. After five weeks, the rats' brains were meticulously removed from the skull.

The RSV used in this study was obtained in powder form from Sigma-Aldrich Chemie GmbH. The RSV was dissolved in ethanol and then diluted in saline to concentrations of 2.5/5 mg/kg, and injected at < 1% ethanol final concentration. Following surgery, the designated treatment groups received daily IP injections of RSV (2.5 or 5 mg/kg) for five consecutive weeks to evaluate its neuroprotective effects. The selected doses were based on the lack of effect on hemodynamic parameters such as baseline blood pressure in previous studies (23, 24).

### 3.3. Induction of the 2-VO Model; Surgical Procedure

The 2-VO model was induced through sequential procedures: First, bilateral carotid artery occlusion was performed in two stages with a 7-day interval between procedures. For each surgery, rats were anesthetized using ip administration of ketamine (80 mg/kg) combined with xylazine (10 mg/kg). Following confirmation of surgical anesthesia, animals were positioned in a supine orientation on a temperature-regulated surgical platform, where the ventral cervical region was carefully shaved and aseptically prepared with povidone-iodine solution. A midline neck incision was made to expose the left and right common carotid arteries for occlusion. The arteries were permanently ligated with 3 - 0 silk suture. Then the wound was closed, disinfected with penicillin, and the animals were returned to their cages after regaining consciousness (24). The Sham group underwent identical procedures except for artery ligation. Animals were monitored until full recovery. The RSV administration began 72 hours post-second surgery after confirming stable health status.

### 3.4. Behavioral Studies

#### 3.4.1. Shuttle Box Test (Passive Avoidance Task)

On postoperative day 27, rats were acclimatized to the shuttle box apparatus. After a 10-second habituation period in the illuminated compartment, the guillotine door was opened. Upon entering the dark compartment, rats received a mild foot shock (0.5 mA, 50 Hz, 2 s). Twenty-four hours later, latency to re-enter the dark compartment and the number of trials required to achieve the learning criterion (e.g., >180 seconds avoidance) were recorded as measures of fear memory retention.

### 3.5. Water Maze Test

#### 3.5.1. Training Protocol (Days 29 - 35)

Rats underwent 4 trials per day to locate a hidden platform in a circular pool divided into quadrants. The duration spent in each quadrant to find the hidden platform was recorded.

#### 3.5.2. Probe Test (Day 35)

The platform was removed to assess spatial memory retention. Time spent in the target quadrant (previous platform location) was quantified (25).

### 3.6. Tissue Preparation for Quantitative Real-time Polymerase Chain Reaction

At the end of the behavioral tests, the animals were euthanized, their brains were immediately removed, and the hippocampi were rapidly dissected on dry ice and stored at -80°C. To analyze the expression of the target genes, the complete RNA was extracted using the FavorPrep^™^ Tissue Total RNA Mini Kit, following the kit's instructions. RNA purity (A260/A280 ratio 1.8 - 2.0) was verified by Picodrop. The isolated RNA was preserved at -80°C until ready for cDNA synthesis. The cDNA synthesis used 1 μg RNA with RevertAid^™^ Reverse Transcriptase. Then, the prepared cDNA was used for real-time polymerase chain reaction (RT-PCR) using SYBR green. Primer sequences for Bax, Bcl-2, Caspase-3, Ras homolog family member A (RhoA), Rho-associated coiled-coil containing protein kinase 2 (ROCK2), CaMKII-α, and NMDAR2B were validated (Table 1). The β-actin was used as the internal control. Thermal cycling conditions and annealing temperatures are detailed in Tables 2. and 3. 

**Table 1.** Primer Sequences (5′-3′) Used in Real-time Polymerase Chain Reaction

Gene	Sequences
**Caspase-3**	
Fwd	5′-GTGGAACTGACGATGATATGGC-3′
Rev	5′-CGCAAAGTGACTGGATGAACC-3′
**Bax**	
Fwd	5′-CGAGCTGATCAGAACCATCA-3
Rev	5′-CTCAGCCCATCTTCTTCCAG-3
**Bcl-2**	
Fwd	TGATTTGACCATTTGCCTGA-3′-5′
Rev	5′-TCTCCACAATGTCAGCTCTC-3′
**RhoA**	
Fwd	5′-CGTTAGTCCACGGTCTGGTC-3′
Rev	5′-CAGCCATTGCTCAGGCAAC-3′
**ROCK2**	
Fwd	5′-GAAGAGCAGCAGAAGTGGGT-3′
Rev	5′-GGCAGTTAGCTAGGTTTGTTTGG-3′
**NMDAR2B**	
Fwd	5-GCCTACAAGCGACACAAGGATG-3′
Rev	5′-TTAGGGTCGGGCTCTGCTCTAC-3′
**CaMK**	
Fwd	5′-CTACGGTTCGCATACTAA-3′
Rev	5′-GCAACCGAACTACTCCTA-3′
**B-actin**	
Fwd	5′-CTCTCTTCCAGCCTTCCTTC-3′
Rev	5′-GGTCTTTACGGATGTCAACG-3′

Abbreviations: Bax, B-cell lymphoma 2-associated X; Bcl-2, B-cell lymphoma 2; RhoA, Ras homolog family member A; ROCK2, Rho-associated coiled-coil containing protein kinase 2; NMDAR2B, N-methyl-D-aspartate receptor subunit 2B.

**Table 2.** Thermal Cycling Conditions

Steps	Temperateure (°C )	Time
**Initial denaturation**	95	10 min
**Denaturation**	95	15 s
**Annealing**	Gene specific (see below)	30 s
**Extension**	72	30 s
**Melt curve**	60 - 95	0.5°C increments

**Table 3.** Annealing Temperateure

Gene	Annealing Temperateure (°C )
**Bcl-2**	58
**Bax**	60
**Caspase-3**	59
**RhoA**	61
**Rcck2**	58
**CaMKII-α**	60
**NMDAR2B**	59

Abbreviations: Bcl-2, B-cell lymphoma 2; Bax, B-cell lymphoma 2-associated X; RhoA, Ras homolog family member A; CaMKII-α, calcium/calmodulin-dependent protein kinase II alpha; NMDAR2B, N-methyl-D-aspartate receptor subunit 2B.

### 3.7. Detection of Calcium/Calmodulin-Dependent Protein Kinase II Alpha and N-methyl-D-aspartate Receptor Subunit 2B Proteins by Western Blot

The hippocampal tissue was lysed and homogenized in a buffer solution. A total of 30 μg protein per lane was separated on 12% SDS-PAGE gels. Subsequently, the proteins were transferred onto a nitrocellulose or PVDF membrane at 56 volts for 2 hours in the transfer buffer. In the blocking step, the membrane was incubated with 5% milk powder in Tris buffer (TBST). Then, the membranes were left to incubate overnight at 4°C with primary antibodies: The CaMKII-α (sc-32288, 1:1000) and NMDAR2B (1:300, ab283731). Protein bands were detected using chemiluminescence reagents (Bio-Rad, Reagents A and B, mixed 1:1) with 5% non-fat milk as a blocking agent and then placed in a gel documentation system.

### 3.8. Statistical Analysis

Statistical analyses were performed with GraphPad Prism software (v.8.0.2). The findings were expressed as the mean ± standard error of the mean (SEM). The MWM test was evaluated using both two-way and one-way analysis of variance (ANOVA). The passive avoidance test, protein, and mRNA expression of BAX, Caspase-3, BCL2, Rho/ROCK2, NMDAR2B, and CaMKII-α were performed using two-way ANOVA followed by Tukey's post-hoc test for multiple comparisons. A P-value < 0.05 was considered statistically significant.

## 4. Results

### 4.1. Results of Spatial Memory and Learning by Morris Water Maze Test

**Figure 1.**
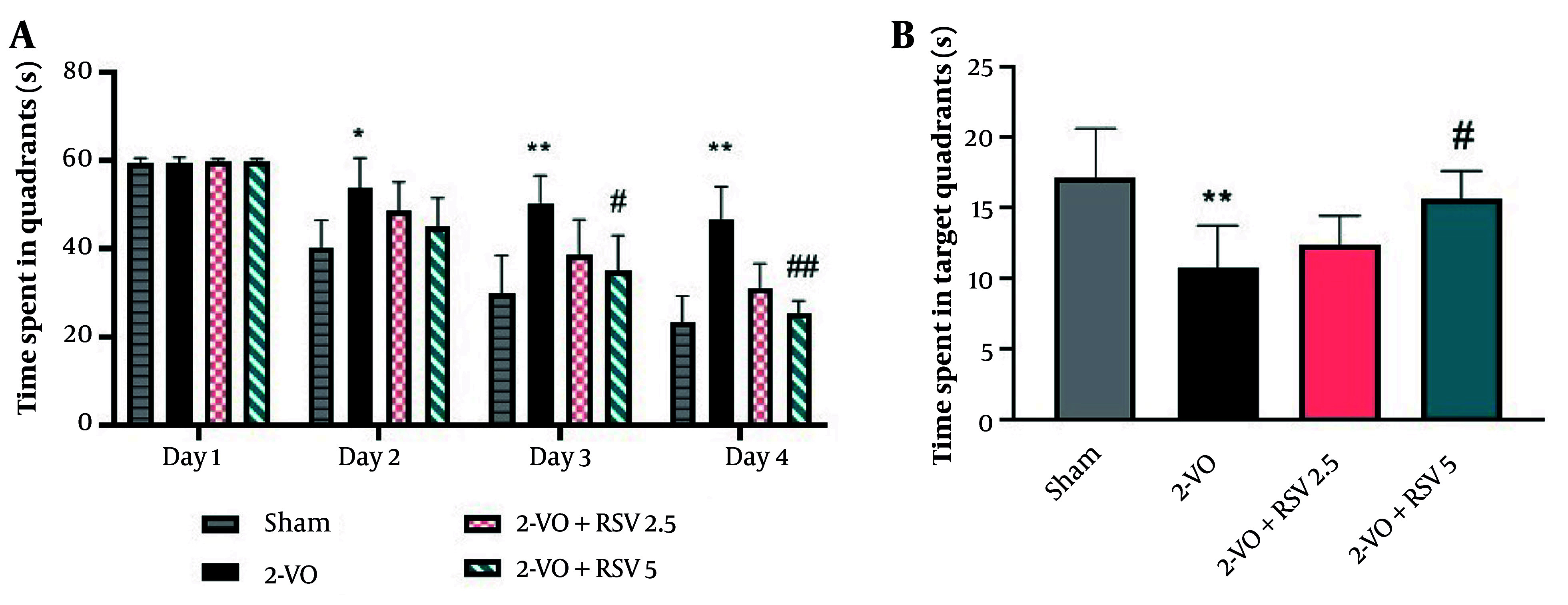
The Morris water maze (MWM) behavioral test: The duration of time spent throughout the training days (A); the duration of the time spent in the target quadrant (B)[data are expressed as mean ± standard error of the mean (SEM); n = 10; differences between groups were conducted by analysis of variance (ANOVA) followed by Tukey's test; * P < 0.05 and ** P < 0.01 vs. Sham and # P < 0.05 and ## P < 0.01 vs. 2-VO].

Spatial learning was evaluated over four consecutive days (time to locate the hidden platform) across four quadrants. As shown in Figure 1A, the time spent to find the platform did not significantly differ among all groups on the first day. On the second day, 2-VO rats demonstrated significantly prolonged escape latency versus Sham (P = 0.0127), indicating early spatial learning deficits. There was no notable difference between the treatment groups and the 2-VO group. This impairment persisted on days 3 and 4, with 2-VO rats showing markedly longer latencies than Sham (P = 0.0065 and P = 0.0312, respectively).

Additionally, on the third day, the 2-VO+RSV5 group showed a notable decrease in time spent compared to the 2-VO group (P < 0.0001), while no significant difference was observed between the rats in the 2-VO+RSV2.5 and 2-VO groups. On the fourth day, a significant reduction in the time spent was observed in the 2-VO+RSV5 group rats compared to the rats in the 2-VO group (P < 0.0001).

The platform was removed 48 hours after the learning trials, and time spent in the target quadrant (NE) was considered a measure of spatial memory. In the 2-VO group rats, swimming time in the target quadrant was significantly lower compared to the Sham group (P = 0.0078, Figure 1B). The RSV at 5 mg/kg (but not 2.5 mg/kg) significantly increased time spent within the target quadrant versus 2-VO (P = 0.0312), suggesting dose-dependent memory rescue. These findings demonstrate that RSV (5 mg/kg) reverses CCH-induced spatial memory deficits, while lower doses show limited efficacy.

### 4.2. Passive Avoidance Memory

**Figure 2.**
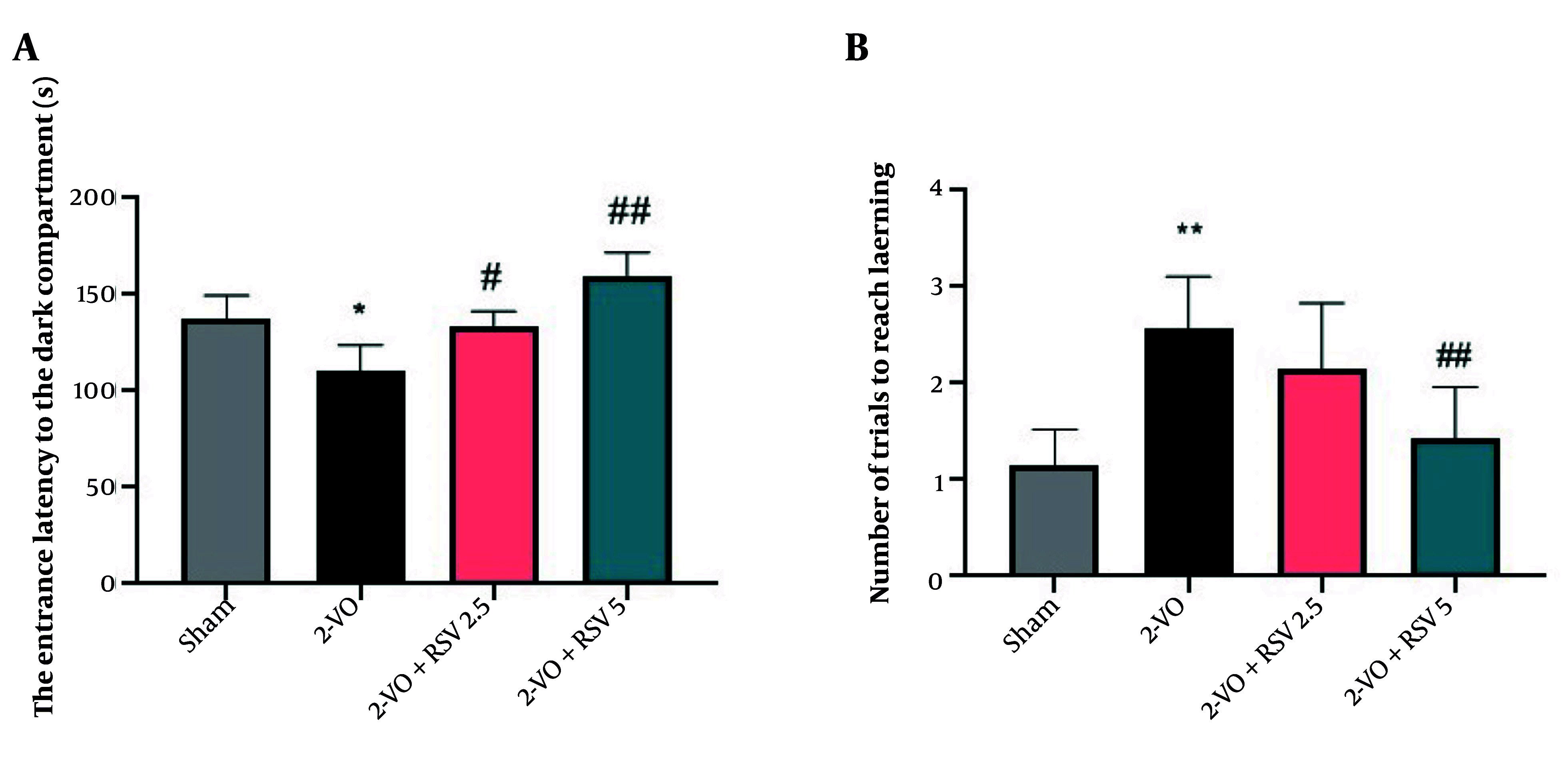
The passive avoidance test: The entrance latency to the dark compartment (A) and number of trials to reach learning in the passive avoidance learning test (B) in the studied groups [data are expressed as mean ± standard error of the mean (SEM); n = 10; differences between groups were conducted by analysis of variance (ANOVA) followed by Tukey's test; * P < 0.05 and ** P < 0.01 vs. Sham and # P < 0.05 and ## P < 0.01 vs. 2-VO].

Figure 2A demonstrates that 2-VO rats showed significantly shorter entrance latency to the dark compartment compared to Sham controls (P = 0.0001), indicating impaired fear memory. Both doses of RSV (2.5 and 5 mg/kg) effectively reversed this deficit, with the 5 mg/kg dose showing greater efficacy (P = 0.0110, P = 0.0012 vs 2-VO). Furthermore, the 2-VO+RSV5 group required fewer trials to reach the learning criterion than 2-VO rats (Figure 2B P = 0.0015), suggesting enhanced fear conditioning.

### 4.3. The mRNA Expression Levels of B-cell Lymphoma 2-associated X, Caspase-3, and BCL2 in Hippocampal Tissue

**Figure 3.**
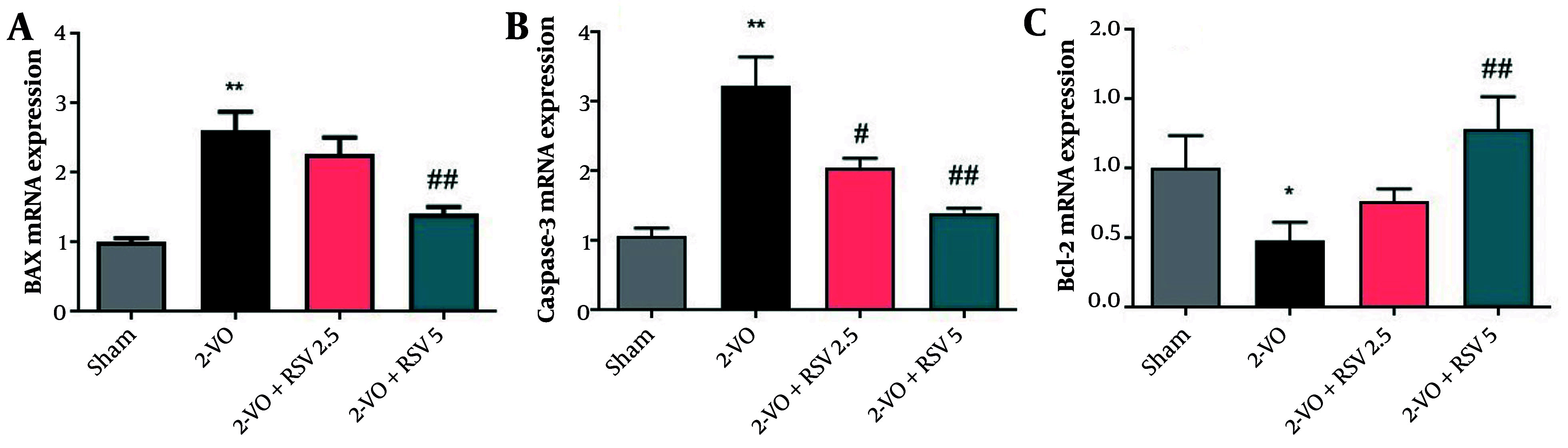
The mRNA expression levels of B-cell lymphoma 2-associated X (BAX) (A), Caspase-3 (B), and BCL2 (C) in hippocampal tissue [data are expressed as mean ± standard error of the mean (SEM); n = 6; differences between groups were conducted by analysis of variance (ANOVA) followed by Tukey's test; * P < 0.05 and ** P < 0.01 vs. Sham and # P < 0.05 and ## P < 0.01 vs. 2-VO].

Our analysis revealed significant dysregulation of apoptotic markers in hippocampal tissue following CCH. Specifically, the 2-VO group demonstrated that the mRNA expression level of BAX was significantly elevated compared to the Sham group (P = 0.0022, Figure 3A). While RSV treatment at 2.5 mg/kg showed a reduction versus the 2-VO group, this change did not reach statistical significance. However, at the 5 mg/kg dose, the decrease was significant in comparison to the 2-VO group (Figure 3A P = 0.0037).

The 2-VO rats exhibited a markedly increased hippocampal Caspase-3 mRNA expression compared to the Sham rats (P = 0.0021). The treatment of RSV at doses of 2.5 and 5 mg/kg led to a notable reduction in Caspase-3 expression in hippocampal tissue relative to the 2-VO group (Figure 3B P = 0.0165, and P = 0.0073, respectively).

Figure 3C demonstrates a significant reduction in BCL2 mRNA expression in the 2-VO group (P = 0.0411). Additionally, the 2-VO+RSV5 group showed a significant increase in BCL2 mRNA expression compared to the Sham group (P = 0.0047).

### 4.4. Hippocampal B-cell Lymphoma 2-associated X/B-cell Lymphoma 2 Ratio

**Figure 4.**
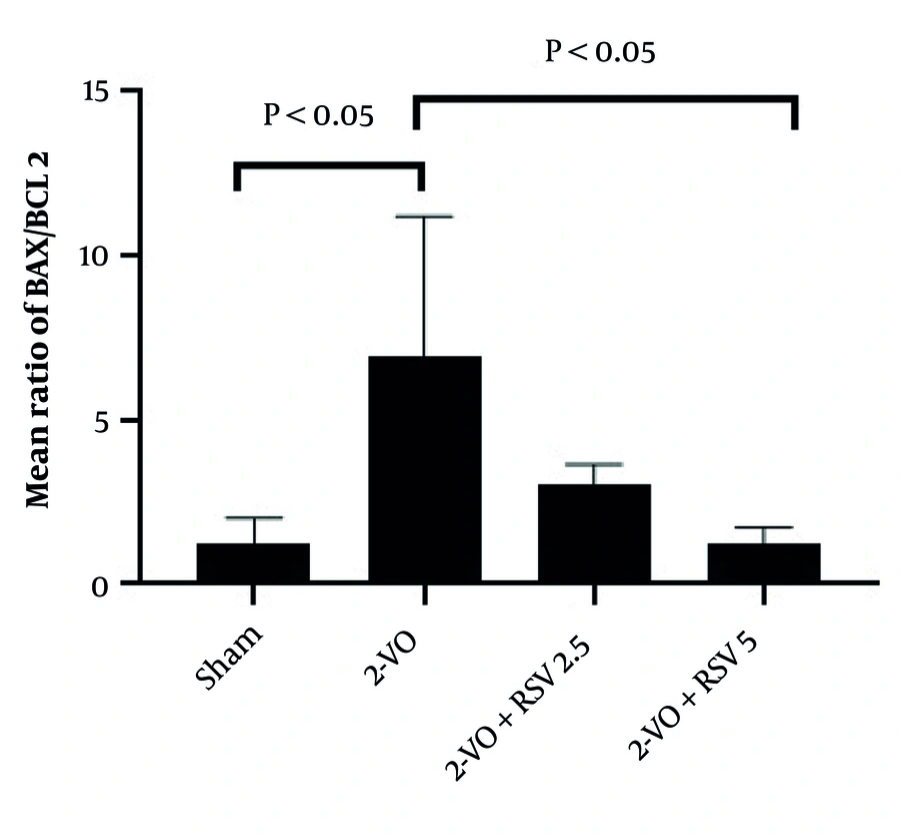
Changes in the B-cell lymphoma 2-associated X (Bax)/B-cell lymphoma 2 (Bcl-2) ratio in hippocampal tissue [data are expressed as mean ± standard error of the mean (SEM); differences between groups were conducted by analysis of variance (ANOVA) followed by Tukey's test].

The change in the Bax/Bcl-2 ratio revealed significant differences among experimental groups. The 2-VO group showed a marked increase in the Bax/Bcl-2 ratio compared to the Sham group (P = 0.0155), indicating a predominance of pro-apoptotic over anti-apoptotic factors (Figure 4). In the treatment groups, RSV at 5 mg/kg (2-VO+RSV5) significantly reduced this ratio compared to the 2-VO group (P = 0.0407). This reduction demonstrates that high-dose RSV shifted the balance toward cell survival mechanisms.

At the molecular level, the elevated Bax/Bcl-2 ratio in the 2-VO group resulted from two concurrent changes: Increased expression of the pro-apoptotic protein Bax coupled with decreased expression of the anti-apoptotic protein Bcl-2. These alterations triggered the mitochondrial apoptosis cascade, ultimately leading to cell death. Conversely, effective doses of RSV reversed this trend. The decreased Bax/Bcl-2 ratio observed in the 2-VO+RSV5 group suggests the compound simultaneously reduced Bax expression while increasing Bcl-2 expression, producing a synergistic inhibitory effect on the apoptosis pathway.

### 4.5. The mRNA Expression Levels of Rho/Rho-associated Coiled-coil Containing Protein Kinase 2 in Hippocampal Tissue

**Figure 5.**
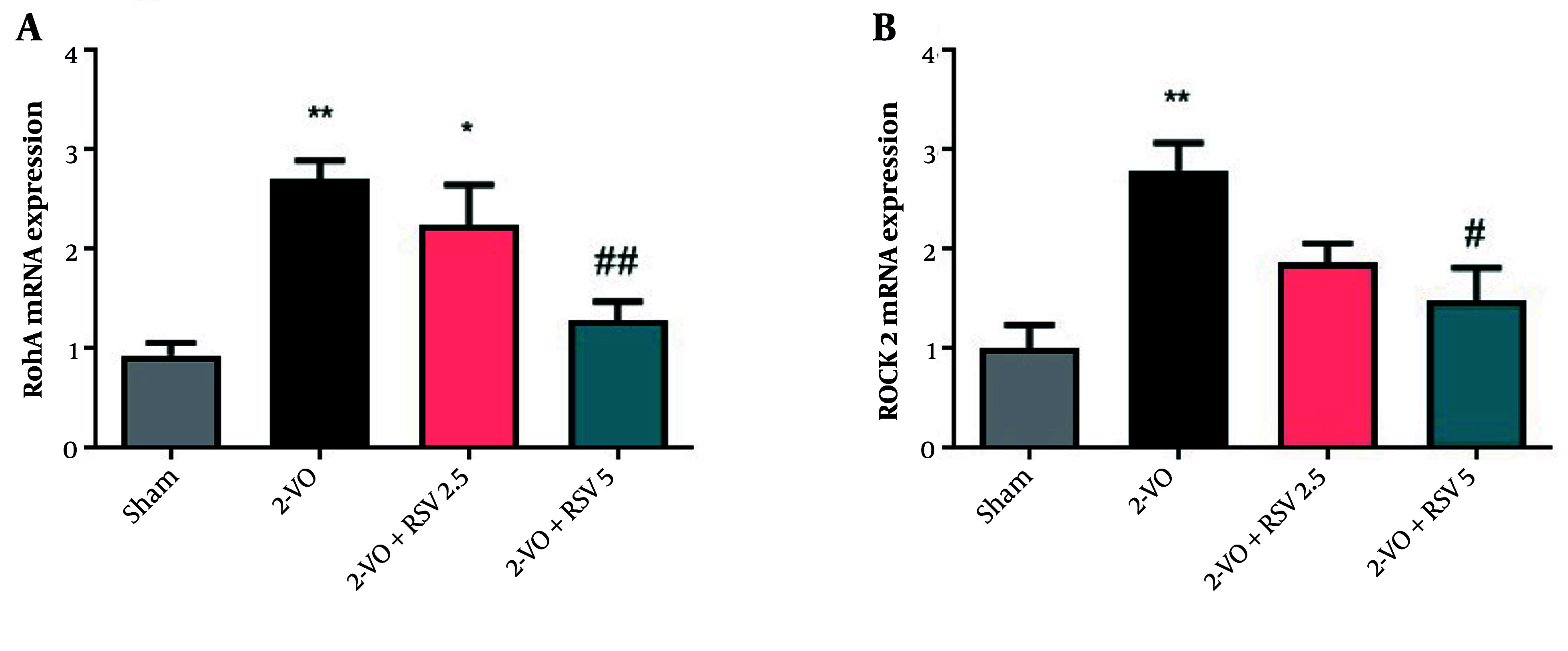
The mRNA expression levels of Ras homolog family member A (RhoA) (A) and Rho-associated coiled-coil containing protein kinase 2 (ROCK2) (B) in hippocampal tissue [data are expressed as mean ± standard error of the mean (SEM); n = 6; differences between groups were conducted by analysis of variance (ANOVA) followed by Tukey's test; * P < 0.05 and ** P < 0.01 vs. Sham and # P < 0.05 and ## P < 0.01 vs. 2-VO].

The Rho/ROCK signaling pathway was markedly activated under 2-VO conditions. Both RhoA and ROCK2 exhibited significant upregulation in comparison to the Sham group (P = 0.0014 for both, Figure 5A and B). The RSV administration at 5 mg/kg effectively normalized RhoA expression (P = 0.0073) and reduced ROCK2 levels (P < 0.05 vs 2-VO group).

At the lower 2.5 mg/kg dose, we observed a significant but partial reduction in RhoA (P = 0.0131), while treatment of animals at this dose led to a decrease in ROCK2 levels that did not reach statistical significance (Figure 5B). 

### 4.6. Protein and mRNA Expression of N-methyl-D-aspartate Receptor Subunit 2B and Calcium/Calmodulin-dependent Protein Kinase II Alpha in Hippocampal Tissue

**Figure 6.**
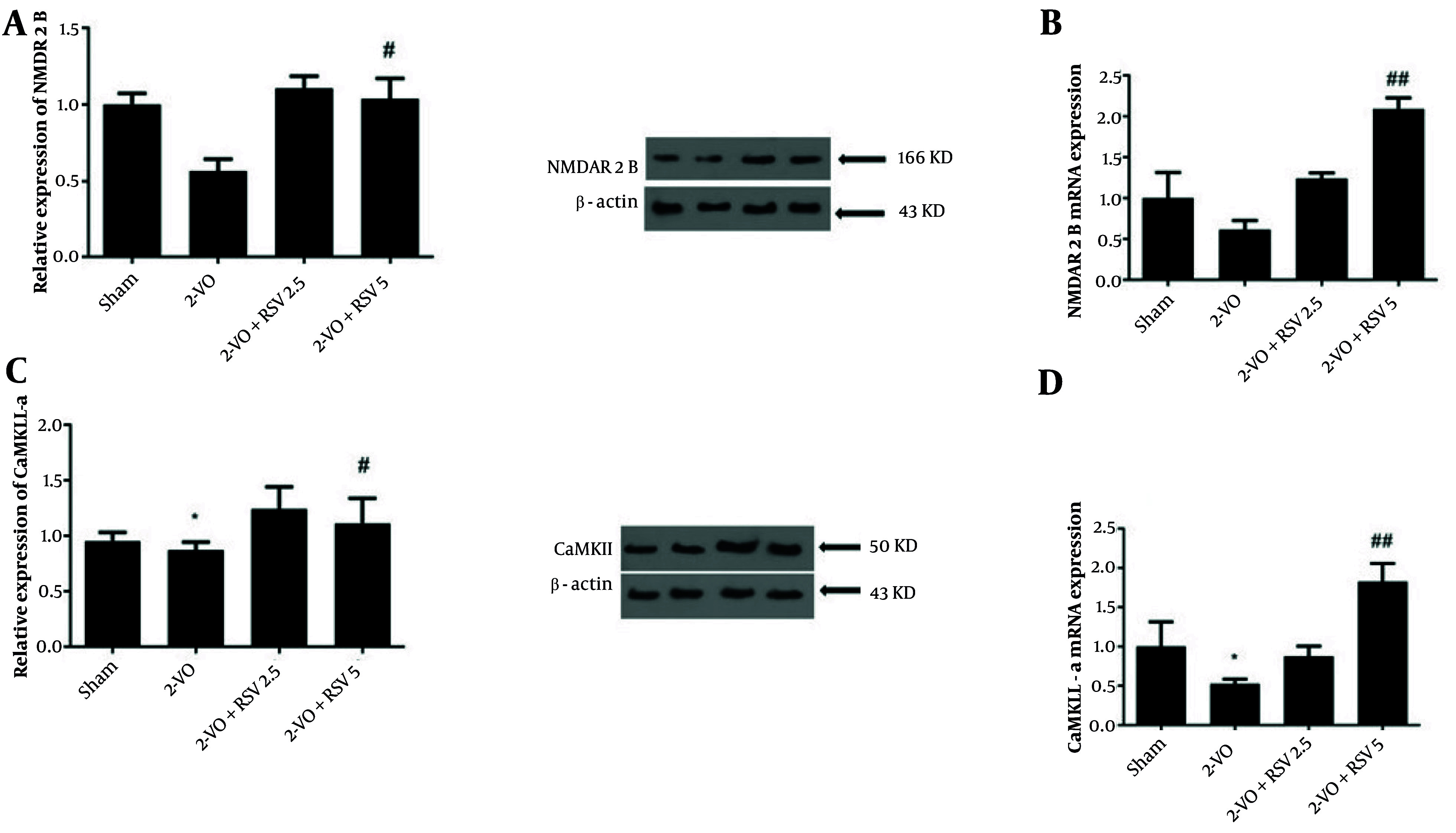
Protein and mRNA expression of N-methyl-D-aspartate receptor subunit 2B (NMDAR2B) (A, B) and calcium/calmodulin-dependent protein kinase II alpha (CaMKII-α) (C, D) in hippocampal tissue [data are expressed as mean ± standard error of the mean (SEM); n = 6; differences between groups were conducted by analysis of variance (ANOVA) followed by Tukey's test; * P < 0.05 vs Sham, # P < 0.05 and ## P < 0.01 vs. 2-VO].

To further investigate the protective effects of RSV in the 2-VO model, we examined hippocampal expression of synaptic proteins. The CCH caused a notable decrease in both NMDAR2B protein levels and mRNA expression in 2-VO mice compared to Sham controls, though these reductions did not reach statistical significance. Treatment with RSV at 5 mg/kg significantly increased NMDAR2B mRNA expression (P = 0.0028) and protein levels (P = 0.0474) relative to untreated 2-VO animals (Figure 6A and B).

For CaMKII-α, 2-VO mice showed significant decreases in both protein and mRNA expression compared to Sham (P = 0.0357, P = 0.0483; Figures 6C and D). The RSV administration at 5 mg/kg effectively reversed these changes, significantly elevating both CaMKII-α protein (P = 0.0497) and mRNA levels (P = 0.0011). While the 2.5 mg/kg dose of RSV showed increasing trends for CaMKII-α expression, these changes were not statistically significant (P > 0.05).

## 5. Discussion

This study aimed to explore RSV's protective role in enhancing memory performance through the reduction of apoptosis and the increase in synaptogenesis in the hippocampus following CCH in rats. In this study, 2-VO group mice exhibited increased Bax and Caspase-3 levels, alongside a reduction in Bcl-2 expression. In fact, one of the pathological features of CCH is neuronal apoptosis, which involves a series of apoptosis-related proteins. Multiple studies have demonstrated that CCH triggers excessive Reactive Oxygen Species (ROS) production, leading to apoptosis and Caspase-3 activation (26). Caspase-3 plays a direct role in apoptosis, and its activation has been reported as essential for initiating apoptotic signaling in many central nervous system diseases, such as VaD (25).

The current investigation clearly demonstrated that CCH significantly increased the levels of apoptotic factors in 2-VO mice, while in the treatment groups, RSV at a dose of 5 mg/kg reduced apoptosis by decreasing the levels of Caspase-3 and Bax and increasing the expression of Bcl-2 in 2-VO mice, resulting in improved memory and learning performance. The RSV, a natural non-flavonoid polyphenol abundant in red grapes, exhibits anti-apoptotic effects in various tissues depending on the dose and duration of use (27), and in our study, these dose-dependent effects were confirmed.

The RSV has been reported to have multiple clinical applications for treating neurodegenerative diseases resulting from CCH, such as Alzheimer's (28) and VaD (29). The RSV increases Sirt1, leading to mitochondrial ATP production and creating a protective property (29). The RSV suppresses cytochrome-C release and mitigates Caspase-3 activation through enhanced activity of antioxidant enzymes [e.g., catalase (CAT), superoxide dismutase (SOD)], effectively blocking apoptosis progression (30).

Consistent with our findings, Zhang et al. demonstrated RSV's efficacy in improving cognitive function in VaD by modulating apoptotic markers (Bax, Caspase-3, Bcl-2) and oxidative stress [malondialdehyde (MDA), SOD] in the hippocampus (29). Taken together, these findings strongly suggest that RSV exerts its neuroprotective effects primarily through modulation of the mitochondrial apoptotic pathway in CCH-induced cognitive impairment. The dose-dependent efficacy observed in our study, particularly the superior effects of 5 mg/kg RSV compared to 2.5 mg/kg, indicates a potential therapeutic threshold for clinical applications.

We hypothesize that RSV may protect against CCH-induced neuronal damage by simultaneously inhibiting multiple apoptotic pathways while activating Sirt1-mediated survival signals. Further studies should investigate the optimal dosing regimen and potential synergistic effects of RSV with other neuroprotective agents to maximize its therapeutic potential for vascular cognitive impairment.

The significant elevation of the Bax/Bcl-2 ratio in the 2-VO group (P < 0.05 vs Sham) reflects a fundamental shift in the apoptotic equilibrium, wherein pro-apoptotic signaling dominates over cellular survival mechanisms. This imbalance occurs through two synergistic molecular events: (1) Upregulation of Bax, which translocates to mitochondrial membranes forming permeability pores, and (2) downregulation of Bcl-2, which normally stabilizes mitochondrial outer membrane integrity. The resulting cytochrome c release initiates Caspase-9/3 activation cascades, consistent with the characteristic neuronal apoptosis observed in CCH models (31).

In this study, we evaluated the effects of CCH on learning, spatial memory, and passive avoidance memory using MWM and shuttle box tests. Consistent with Wang et al., CCH disrupts spatial learning and memory in the MWM test (32). Bayat et al., using the shuttle box test, clearly indicated that CCH leads to impaired passive avoidance memory (33). These studies have reported that CCH plays a significant role in the progression of cognitive impairments in VaD, contributing to neuronal damage and memory deficits.

In the present study, the disruption in spatial and passive avoidance memory in 2-VO rats was well manifested by spending less time in the target quadrant to find the platform and the entrance latency to the dark compartment. The findings of the current study are consistent with previous reports (34). Notably, our results demonstrated that RSV (5 mg/kg) effectively reversed these spatial and passive avoidance memory deficits. Together, these behavioral results demonstrate that RSV (especially at 5 mg/kg) effectively reverses CCH-induced memory deficits. This aligns with its known antioxidant and anti-apoptotic properties, as observed in our molecular analyses. Future studies could explore optimal treatment durations or combination therapies with RSV.

Notably, high-dose RSV (5 mg/kg) not only normalized but functionally reversed this apoptotic predisposition, reducing the Bax/Bcl-2 ratio compared to the 2-VO group. This suggests RSV operates through a dual mechanism: Transcriptional repression of Bax via p53 inhibition (35) while concurrently enhancing Bcl-2 expression through SIRT1-mediated deacetylation of FoxO transcription factors (36). The dose-dependent efficacy further supports the pharmacological specificity of this regulation.

Neuronal recovery, particularly in the hippocampus, depends on cortical plasticity mechanisms including synaptic remodeling (37). Synaptogenesis is essential for neuroplasticity in CCH (38). This study found that the expression levels of CaMKII-α and NMDA2B proteins were markedly lower in the 2-VO group. As glutamatergic ion channels, NMDA receptors are critical for synaptic plasticity and memory processes (39). The elevated NMDA2B levels in the hippocampus of the Sham group suggest its significant role in memory and information processing within this brain region. The calcium-dependent kinase CaMKII-α, enriched in postsynaptic regions, mediates NMDA receptor-dependent learning and memory. It has been shown that the levels of CaMKII-α and NMDAR expression can almost represent the status of synaptogenesis (40).

A study by Niu et al. reported that the lowest levels of CaMKII-α and NMDA2B were observed in the 2-VO group, indicating the loss of synapses due to CCH, which is consistent with the results of the current study (41). It has been reported that learning and memory are the result of changes in synapses identified by patterns of neuronal activity of NMDA receptors (42). In the present study, the upregulation of CaMKII-α and NMDAR2B by RSV suggests activation of Ca^2+^/CaMKII signaling, though direct causal evidence requires further investigation. This dual action on apoptosis and synaptic proteins (CaMKII-α/NMDAR2B) provides compelling evidence for RSV's multimodal neuroprotection. It has been reported that Ca^2+^/CaMKII plays an important role in regulating neuroplasticity and the pathogenesis of cognitive impairments (43). The RSV has effects on calcium signaling, activating CaMKII by increasing intracellular calcium (44). It has also been reported that under ischemic conditions, CaMKII is activated by binding to Ca^2+^/calmodulin and leads to the transfer of activated CaMKII to postsynaptic sites, which play a role in synaptic plasticity (45, 46). Together, these data imply that RSV rescues CCH-induced synaptic dysfunction primarily through CaMKII-α/NMDAR2B modulation. While our findings align with known mechanisms of RSV, further studies should explore its long-term effects on synaptic ultrastructure.

The last part of the study revealed a notable upregulation of RhoA/ROCK2 expression in the hippocampus of CCH mice, while treatment with RSV significantly reduced the mRNA expression of RhoA/ROCK2. The ROCK2 is a downstream target of Rho and is mainly present in regions such as the brain cortex (47). It has been reported that targeting the Rho/ROCK signaling pathway can be a useful strategy for neural regeneration in neurodegenerative diseases (48). The present study results demonstrated that Rho/ROCK may play an important role in regulating synapses in the hippocampus, as it was clearly shown that RSV treatment was able to reduce the expression of ROCK2 and Rho, which is consistent with the increase in the expression of CaMKII-α and NMDA2B in the hippocampus of CCH mice. We hypothesize that Rho/ROCK inhibition potentially creates a permissive environment for synaptic recovery. Future studies should explore whether pharmacological Rho/ROCK inhibition potentiates RSV's effects on synaptic proteins.

The restoration of Bax/Bcl-2 homeostasis correlates strongly with our observed neurobehavioral improvements, positioning this ratio as both a predictive biomarker and therapeutic target for vascular cognitive impairment.

### 5.1. Conclusions

This study demonstrates that RSV at higher doses (5 mg/kg) effectively mitigates CCH-induced cognitive impairments and hippocampal neuronal damage, with attenuated effects observed at lower doses (2.5 mg/kg). The neuroprotective mechanisms appear to involve dual pathways: (1) Suppression of apoptosis (via Bax/Caspase-3 downregulation and Bcl-2 upregulation) and (2) enhancement of synaptogenesis (through CaMKII-α/NMDAR2B restoration and Rho/ROCK pathway inhibition). These dose-dependent effects highlight RSV’s potential as a multi-target therapeutic agent for vascular cognitive impairment, warranting further clinical investigation into its optimal dosing and long-term efficacy.

## Data Availability

The dataset presented in the study is available on request from the corresponding author during submission or after publication.
